# Complication of *Salmonella* Bacteremia in a Case of Treated Fungal Endophthalmitis

**DOI:** 10.1155/2012/198637

**Published:** 2012-02-13

**Authors:** J. Malathi, M. Sowmiya, Vikas Khetan, K. Lily Therese, H. N. Madhavan

**Affiliations:** ^1^Department of Microbiology, L&T Microbiology Research Centre, Sankara Nethralaya, New 41 (Old No. 18) College Road, Chennai 600 006, India; ^2^Vitreo-Retinal Department, Sankara Nethralaya, Chennai, India

## Abstract

This is to report a case of bacteremia caused by *Salmonella typhi* in a treated unilateral fungal endogenous endophthalmitis in an 18-year-old male from one of the South Asian countries. Microbiological and molecular investigations were carried out on the eviscerated material and routine blood culture was carried out. Direct examination of eviscerated material revealed the presence of fungal filaments. However, *Salmonella typhi* was isolated from both specimens, which was confirmed by Polymerase chain reaction targeting the *16SrRNA* gene, sequencing, and random amplification of polymorphic DNA showed that they belonged to the same clone. The presence of *Salmonella* bacteremia in a treated unilateral fungal endophthalmitis, among young adult patients is rare and systemic symptoms should be investigated.

## 1. Case Description

An eighteen-year-old student from one of the South Asian countries reported with a sudden blurring of vision and redness in the right eye (OD) for which he sought a local ophthalmologist's opinion. The patient underwent a lensectomy along with vitrectomy, elsewhere for suspected endophthalmitis. The vitreous sample was suggestive of fungal infection. He received intravitreal injection of Amphotericin B along with vancomycin and ceftazidime at the time of first surgery elsewhere. After the operation, he had perception of light. Two days after the surgery, a repeat intravitreal injection of Amphotericin B and antibiotics were administered, following which his vision did not improve. He was then referred to our institute, where on examination, he was found to have no light perception. The intraocular contents could not be seen because of anterior chamber being full of exudates. Ultrasound revealed the presence of total retinal detachment with shallow choroidal detachment.

Prior to the onset of ocular complication the patient had a history of diarrhoea for 10 days. The patient was advised evisceration of the right eye. At this point, the patient had fever touching up to 101°F, cold, cough, and vomiting. Once the fever subsided, the evisceration was performed under general anaesthesia. Following the evisceration, patient had intermittent fever with high Alanine aminotransferase (ALT) levels. The patient was tested negative for HIV. Clinical examination ruled out the presence of pulmonary tuberculosis.

A bacterial and fungal culture was put up for eviscerated material and the sample was subjected to direct examination using KOH/Calcofluor for fungus and Gram-s stain for bacteria. A few septate fungal filaments were seen in the KOH/Calcofluor stain, and a few Gram-negative bacilli along with pus cells were seen in the Gram-stained smear. The blood sample was processed for bacterial and fungal culture using the BACTEC (Becton, Dickinson and Company) system. Clean catch mid stream urine was subjected to conventional semiquantitative method of isolation bacteria.

**Figure 1:**
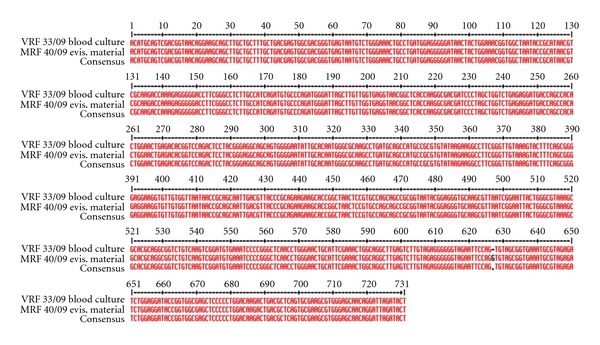
Multalin results showing 100% correlation of 16s rRNA gene of *Salmonella typhi* isolated from blood culture and eviscerated material.

**Figure 2:**
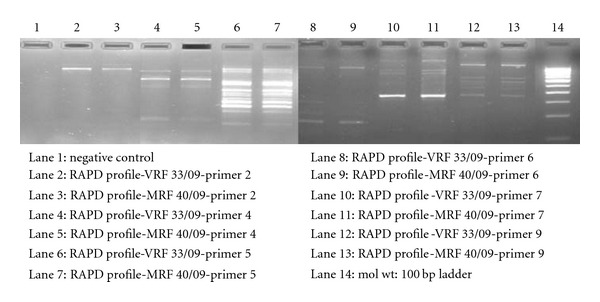
Representative results of random amplification of polymorphic DNA finger printing profiles of *Salmonella typhi* isolated from blood culture and eviscerated material by RAPD PCR using primers 2, 4, 5, 6, 7, and 9.

After three days of incubation, *Salmonella typhi* was isolated from the eviscerated material and blood specimen, whereas the urine culture was negative for pyogenic bacteria. The isolated organism, from both the specimen, was subjected to a nested PCR amplification using primers targeted against the *16S rRNA* gene following the protocol described by us earlier [1]. The first round of PCR amplified products was then subjected to a DNA sequencing to find the sequence homology among the isolates. The sequenced data was analysed using the BLAST search tool and were aligned using the MultAlin software. The DNA sequences of organisms isolated from the blood and eviscerated material confirmed *Salmonella typhi *with 98% identity. The Multalin picture of *16SrRNA* of the isolates was identical as shown in Figure 1. The DNA extracts of the isolates were also subjected to a RAPD (Bangalore Genei Pvt. Ltd) analysis to determine their clonal origin which showed that they belonged to the same clone as shown in Figures 2(a) and 2(b). The patient was treated with systemic antibiotics based on the sensitivity report.

## 2. Discussion

Endogenous endophthalmitis is a complication of sepsis and is most commonly reported among the immuno-compromised individuals. A unilateral presentation is frequently observed compared to a bilateral presentation [2]. The Gram-negative bacilli, which causes foci of infection in some part of the body, is known to progress and cause endogenous endophthalmitis if the immune status of the individual is compromised. Endogenous endophthalmitis, due to *Salmonella typhimurium,* is reported in a 1-year-old child [3] and metastatic endophthalmitis, caused by *Salmonella typhimurium,* in a leukemic patient is reported by Weinstein et al. [4]. Endogenous endophthalmitis, due to *Salmonella choleraesuis* in an HIV-positive patient, is reported by Yodprom et al. [5]. This is the first report of unilateral endogenous endophthalmitis associated with fungus and *S. typhi* in an HIV-negative individual. Bilateral endogenous panophthalmitis is reported by Arora et al. [2]. In this case, the right eye was already infected with fungus in the form of fungal endophthalmitis, for which the patient underwent treatment. The subsequent systemic infection due to *S. typhi* resulted in further complications. The present case is a rare complication of *Salmonella *bacteremia in a treated fungal endophthalmitis patient. *Salmonella typhi *association with endogenous endophthalmitis was reported infrequently in very young [6] and adult patients with predisposing chronic systemic disease.
